# Whipple Procedure vs. Distal Pancreatectomy: A Study on the Efficacy, Survival Rates, and Complication Rates in Patients With Pancreatic Cancer

**DOI:** 10.7759/cureus.81091

**Published:** 2025-03-24

**Authors:** Hamza Siddique, Sabaina Arshad Tarar, Ahmed Salman Majeed, Muhammad Hamza, Mueez Saleem Raja, Muhammad Haider Naqvi, Muhammad Zarar, Fizza Chudhary, Bilal Qammar

**Affiliations:** 1 Internal Medicine, Social Security Hospital, Faisalabad, PAK; 2 General Surgery, Shalamar Hospital, Lahore, PAK; 3 Hepatobiliary and Liver Transplant Surgery, Pakistan Kidney and Liver Institute, Lahore, PAK; 4 General Surgery, Muzaffarabad General Hospital, Muzaffarabad, PAK; 5 Medicine and Surgery, Queen Mary University, London, GBR; 6 General Surgery, District Head Quarters (DHQ) Hospital, Faisalabad, PAK; 7 Emergency Medicine, Novocare International Hospital and Research Center, Sargodha, PAK

**Keywords:** complication rates, distal pancreatectomy, efficacy, pancreatic cancer, survival rates, whipple procedure

## Abstract

Introduction: Pancreatic cancer remains one of the most aggressive and challenging malignancies to treat, with limited options for curative interventions.

Objective: The basic aim of the study is to find and compare the efficacy, survival rates, and complication rates of the Whipple procedure versus distal pancreatectomy in patients with pancreatic cancer.

Methodology: This retrospective observational study was conducted at Pakistan Kidney and Liver Institute, Lahore. Patients were enrolled from November 2018 to November 2019. Data were collected from 30 patients. Data were collected from the patient's medical records to evaluate and compare the outcomes of the two surgical approaches. The 30 patients were categorized into two equal groups based on the surgical intervention they received.

Results: The mean age was comparable between groups, with an overall mean of 59.5 years. Tumors in Group A (Whipple procedure) were larger on average (3.8 ± 1.2 cm) compared to Group B (distal pancreatectomy; 3.2 ± 1.1 cm). Lymph node involvement was observed in 16 (53.3%) patients overall, with Group A showing a slightly higher rate (nine, 60%) compared to Group B (seven, 46.7%). Additionally, Group A had a higher mean number of lymph nodes removed (18) than Group B (15), while comorbidities were reported in 11 (36.7%) patients overall. The study found that the readmission rate was higher in Group A at three (20%), compared to two (13.3%) in Group B, reflecting the more complex and challenging postoperative course associated with the Whipple procedure.

Conclusion: The Whipple procedure, performed for pancreatic head tumors, offers better long-term survival but carries a higher risk of complications, including delayed gastric emptying and pancreatic fistulas. In contrast, distal pancreatectomy, used for tumors in the body or tail, is associated with lower surgical complexity and fewer complications but may have less favorable survival outcomes due to the later-stage presentation of these tumors. Additionally, the frequent need for splenectomy in distal pancreatectomy increases the risk of postoperative infections.

## Introduction

Pancreatic cancer remains one of the most aggressive and challenging malignancies to treat, with limited options for curative interventions. Surgical resection remains the mainstay of treatment since it provides the only curative approach to the disease in particular circumstances. Of the surgical operations, the Whipple procedure (pancreaticoduodenectomy) and distal pancreatectomy are performed according to the site and grade of tumors [1]. The Whipple procedure seems appropriate for tumors in the head of the pancreas, while distal pancreatectomy is used for tumors in the body and tail [2]. The Whipple procedure is a major surgery with the resection of the pancreatic head, duodenum, gallbladder, and bile duct, followed by a reconstruction of the digestive pathway. The Whipple procedure is widely recognized as one of the most complex abdominal surgeries, with reported high morbidity and mortality rates [3]. However, the Whipple procedure is still considered a potentially curative operation for patients with pancreatic head malignancies because it confers a survival advantage in patients who are surgical candidates. On the other hand, distal pancreatectomy, where the body of the pancreas and the tail are resected, is less likely to involve much more anatomical dissection [4]. Distal pancreatectomy, although technically easier and less complex than the Whipple procedure, is still associated with risks such as postoperative pancreatic fistulas and splenic complications [5]. This procedure is mainly used for tumors arising at the end of the pancreas body and carries different overall survival and recurrence rates. Therefore, knowledge of the comparative performance, survival benefits, and complication risks associated with these two operations is essential in decision-making [6]. Each procedure is significant in pancreatic cancer treatment, but their outcomes primarily depend on tumor location rather than a direct comparison. Therefore, research that compares the results of these two surgeries will be useful in determining their usefulness, safety, and consequences for patients [7]. Pancreatic cancer control by the Whipple procedure and distal pancreatectomy depends on the considerations of disease biology, tumor stage, and margins of resection. Obtaining negative surgical margins is one of the major factors predicting survival in both surgeries. These trends of survival are further determined by other factors such as staging of the tumor, node involvement, and presence of allied disease [8]. The Whipple procedure involves more extensive resections than simpler measurements such as the pylorus-preserving procedure, but patients suffering from localized pancreatic head cancers have had better survival rates with this operation [9]. Distal pancreatectomy is less invasive, and for this reason, it is done in patients with tumors that might be biologically different and therefore affect overall survival. Another important factor used in evaluating such surgeries is the rate of complications. Both surgeries have their complications, for example, postoperative infection, delayed gastric emptying, pancreatic fistulas, and others [10]. The Whipple procedure offers an overall higher rate of complications because of the extensive nature of the surgery; however, distal pancreatectomy, which is considered less hazardous, carries a higher risk of fistula formation [11].

Pancreatic cancer outcomes are critically influenced by surgical approach, yet the comparative efficacy of the Whipple procedure (for head tumors) and distal pancreatectomy (for body/tail tumors) remains understudied. While both aim for curative resection, differences in tumor biology, surgical complexity, and complication profiles may influence survival and recovery. This study directly compares these approaches to inform clinical decision-making.

## Materials and methods

Study design and setting

This retrospective observational study was conducted at the Pakistan Kidney and Liver Institute, Lahore, Pakistan. Patients were enrolled from November 2018 to November 2019. Data were collected from 30 patients.

Participant selection

Patients with histologically confirmed pancreatic cancer who met the criteria for curative resection based on preoperative imaging (CT, MRI) and multidisciplinary team assessment were included. Patients were included if they had localized, resectable (T1-3, N0-1, M0) pancreatic adenocarcinoma confirmed by histopathology. The tumor stage was classified using the 8^th^ edition of the American Joint Committee on Cancer - Tumor, Node, Metastasis (AJCC TNM system). Additionally, complete medical records, including preoperative, intraoperative, and postoperative data, were required for inclusion. Conversely, patients were excluded if they had metastatic or unresectable pancreatic cancer, incomplete medical records, or if they underwent palliative surgical procedures or other non-curative interventions.

Data collection

Data were collected from patient medical records to evaluate and compare the outcomes of the two surgical approaches. The 30 patients were categorized into two equal groups based on the surgical intervention they received. Group A included patients treated with the Whipple procedure, which is indicated for tumors in the pancreatic head. Group B consisted of patients treated with distal pancreatectomy, typically performed for tumors in the pancreatic body or tail. This distribution favored an equitable comparison of the results between both types of surgery. Demographic and baseline clinical characteristics that included age, sex, and comorbid conditions were also documented to check the similarity in the groups and control for possible confounding factors. Some other details about the operation, for example, the time for surgery, the amount of hemorrhage, and information about the lymph nodes removed were also recorded. Outcomes were assessed according to resection status (R0 and pathological TNM stages), survival (one-, two-, and five-year overall and progression-free survival (PFS)), and morbidity. Pancreatic fistulas, infection, and delayed gastric emptying were carefully documented in the postoperative period to evaluate the safety of the two operations.

Surgical technique

The Whipple procedure involved the removal of the pancreatic head, part of the duodenum, gallbladder, and bile duct, along with reconstruction to restore digestive continuity. This complex procedure is often associated with significant perioperative challenges. Distal pancreatectomy, on the other hand, entailed the removal of the pancreatic body and tail, often accompanied by splenectomy due to anatomical proximity.

Statistical analysis

Data were analyzed using IBM SPSS Statistics software for Windows, version 26 (IBM Corp., Armonk, NY). Continuous variables (e.g., tumor size, lymph nodes removed, median PFS, and hospital stay) were summarized using means and standard deviations and compared between groups using an independent t-test. Categorical variables (e.g., survival rates, complication rates, and readmission rates) were expressed as frequencies and percentages and analyzed using the chi-square test (χ² test). If an expected cell frequency was <5, Fisher’s exact test was applied instead of the chi-square test. Non-parametric tests (Mann-Whitney U) were used for skewed data (e.g., median PFS).

Statistical significance was set at p < 0.05. Test statistics (t-values for t-tests, χ²-values for chi-square tests) and p-values are reported in the results tables.

## Results

The study included 30 patients, divided equally between the Whipple procedure (Group A) and distal pancreatectomy (Group B). The mean age was comparable between groups, with an overall mean of 59.5 years. The mean tumor size was larger in Group A (3.8 ± 1.2 cm) than in Group B (3.2 ± 1.1 cm), but this difference was not statistically significant (t = 1.43, p = 0.164). Lymph node involvement was observed in 16 (53.3%) patients overall, with Group A showing a slightly higher rate (nine, 60%) compared to Group B (seven, 46.7%). Additionally, Group A had a higher mean number of lymph nodes removed (18) than Group B (15), while comorbidities were reported in 11 (36.7%) patients overall (Table 1).

**Table 1:** Demographic and baseline characteristics of the patients t-values are reported for continuous variables (mean age, mean tumor size, and mean lymph nodes removed) using an independent t-test. χ²-values are reported for categorical variables (male-to-female ratio, lymph node involvement, comorbidities) using a chi-square test. p-values < 0.05 are considered statistically significant. N/A: no statistical test was performed for the number of patients and tumor location (structural difference).

Characteristic	Group A (Whipple procedure)	Group B (Distal pancreatectomy)	Total (n=30)	Test statistic	P-value
Number of patients (n)	15	15	30	-	-
Mean age (years)	59.01 ± 2.35	58.90 ± 3.11	59.5 ± 5.46	T = 0.11	0.916
Male-to-female ratio	02:01	02:01	02:01	Χ² = 0.00	1
Mean tumor size (cm)	3.8 ± 1.2	3.2 ± 1.1	3.5 ± 1.2	T = 1.43	0.164
Lymph node involvement (%)	9 (60%)	7 (46.7%)	16 (53.3%)	Χ² = 0.54	0.464
Mean lymph nodes removed	18 ± 4	15 ± 4	16.5	T = 1.92	0.07
Comorbidities (%)	6 (40%)	5 (33.3%)	11 (36.7%)	Χ² = 0.14	0.705
Tumor location	Head of the pancreas	Body/tail of the pancreas	-	-	-

The study revealed that R0 resection, indicating negative surgical margins, was achieved in 13 (86.7%) patients in Group A and 12 (80%) patients in Group B. Group A had a higher mean number of lymph nodes removed (18) compared to Group B (15), reflecting the more extensive nature of the Whipple procedure (Table 2).

**Table 2:** Efficacy of surgical resection t-values are reported for continuous variables (mean lymph nodes removed) using an independent t-test. χ²-values are reported for categorical variables (R0 resection, positive nodes) using a chi-square test. p-values < 0.05 are considered statistically significant.

Parameter	Group A (Whipple procedure)	Group B (Distal pancreatectomy)	Test statistic	p-value
R0 resection (%)	13 (86.7%)	12 (80%)	χ² = 0.24	0.624
Mean lymph nodes removed	18 ± 4	15 ± 4	t = 1.92	0.07
Positive nodes (%)	6 (33.3%)	4 (26.7%)	χ² = 0.17	0.677

In Table 3, survival outcomes indicate that Group A had slightly higher overall survival rates compared to Group B at one year (13 (86.7%) vs. 12 (80%)), two years (10 (66.7%) vs. nine (60%)), and five years (six; 40%) vs. five (33.3%)). The median PFS was also longer in Group A (18 months) compared to Group B (15 months). Complications were more frequent in Group A, with overall complications observed in 10 (66.7%) patients, compared to eight (53.3%) patients in Group B. Pancreatic fistulas were slightly more common in Group B (five (33.3%)) than in Group A (four (26.7%)), while delayed gastric emptying was more prevalent in Group A (three (20%) vs. one (6.7%)). Postoperative infections were also higher in Group A (three (20%)) compared to Group B (two (13.3%)).

**Table 3:** Survival outcomes and complications U-values are reported for median PFS using non-parametric tests (Mann-Whitney U). χ²-values are reported for categorical variables (survival rates, complication rates) using a chi-square test. Fisher’s exact test was used for delayed gastric emptying due to low expected counts. p-values < 0.05 are considered statistically significant. PFS: progression-free survival

Parameter	Group A (Whipple procedure)	Group B (Distal pancreatectomy)	Test statistic	p-value
One-year overall survival	13 (86.7%)	12 (80%)	χ² = 0.24	0.624
Two-year overall survival	10 (66.7%)	9 (60%)	χ² = 0.10	0.756
Five-year overall survival	6 (40%)	5 (33.3%)	χ² = 0.14	0.705
Median PFS (months)	18	15	U = 97.5	0.21
Complications				
Pancreatic fistulas	4 (26.7%)	5 (33.3%)	χ² = 0.16	0.69
Delayed gastric emptying	3 (20%)	1 (6.7%)	Fisher’s exact	0.598
Postoperative infections	3 (20%)	2 (13.3%)	χ² = 0.24	0.624
Overall complications	10 (66.7%)	8 (53.3%)	χ² = 0.56	0.456

The study found that the readmission rate was higher in Group A at three (20%), compared to two (13.3%) in Group B, reflecting the more complex and challenging postoperative course associated with the Whipple procedure. No perioperative mortality was reported in either group, indicating the safety of both surgical approaches with proper perioperative care. Patients in the Whipple procedure group had a significantly longer hospital stay (14 ± 3.5 days) compared to the distal pancreatectomy group (11 ± 2.8 days, t = 2.59, p = 0.015), indicating a statistically significant difference (Table 4).

**Table 4:** Readmission and mortality rates t-values are reported for continuous variables (mean hospital stay) using an independent t-test. χ²-values are reported for categorical variables (readmission rate) using a chi-square test.

Parameter	Group A (Whipple procedure)	Group B (Distal pancreatectomy)	Test statistic	p-value
Readmission rate (%)	3 (20%)	2 (13.3%)	χ² = 0.24	0.624
Perioperative mortality	0%	0%	-	-
Mean hospital stay (days)	14 ± 3.5	11 ± 2.8	t = 2.59	0.015

## Discussion

This study provides a comparative analysis of the outcomes of the Whipple procedure and distal pancreatectomy in patients with pancreatic cancer, focusing on efficacy, survival rates, and complication profiles. Both procedures are valuable in the management of pancreatic cancer, and knowledge of their strengths and weaknesses provides increased patient care value. The study highlighted the high frequency of R0 resection both among the Whipple procedure and distal pancreatectomy groups, the latter reporting slightly higher 13 patients (86.7%) and 12 patients (80%) of R0 resection, respectively [12]. This aligns with previous studies, which reported R0 resection rates of 85% and 78% for Whipple and distal pancreatectomy, respectively, emphasizing the importance of achieving negative surgical margins for long-term survival [13]. Evaluating negative margins is critical to the disease’s long-term management, and both procedures assist in this manner, according to the findings. The higher R0 resection rate in the Whipple procedure (86.7%) compared to distal pancreatectomy (80%) may be due to the more extensive resection of surrounding structures. However, differences in tumor biology and stage at presentation may also contribute to this finding. Despite this, the slightly inferior outcomes of distal pancreatectomy might be attributed to the mere biological behavior of tumors located in the body and tail of the pancreas or technical resectability in these regions [14]. Regarding the survival results, it is possible to state the relations with the existing data, which point to somewhat better long-term survival in the group of patients who underwent the Whipple procedure. At the five-year follow-up, the patient survival of Group A was six patients (40%), while for Group B the survival rate was five patients (33.3%); however, this difference was not statistically significant (χ² = 0.14, p = 0.705) [15]. Similar trends have been reported, where five-year survival rates were 38% for Whipple and 30% for distal pancreatectomy, reinforcing the impact of tumor location and biology on survival rather than surgical technique alone [16]. This also may be attributed to variations in the tumor characteristics and probably the stage, since tumors of the head of the pancreas frequently present at an earlier stage due to symptoms such as jaundice. Extra-cervical tumors often present relatively harmless symptoms until later stages, and these late-stage symptoms could harm the survival rate even if affected patients undergo surgery [17]. Progression-free survival was similar, and a comprehensive outcome benchmarking revealed effective short- to medium-term disease management in both arms. A retrospective study found that median PFS was 16 months for Whipple and 14 months for distal pancreatectomy patients, closely matching the findings of this study (18 vs. 15 months) [18]. It was expected that the Whipple procedure would also bear more postoperative complications, which were more frequent and severe as compared to the other groups [19]. Specifically, the rate of delayed gastric emptying was higher in the Whipple group (20% vs. 6.7%), which has been documented in other studies, where Whipple patients had delayed gastric emptying rates of 18% to 22% [20]. This study found higher rates of pancreatic fistulas in the distal pancreatectomy group (33.3% vs. 26.7%), which is in line with previous research, where distal pancreatectomy had a fistula rate of 30% to 35%, compared to 20% to 25% in Whipple patients [21]. Delays in gastric emptying and infections in patients in this group caused longer hospital stays and more hospital readmissions accordingly. Other low morbidity and mortality rates observed in this study include liver mobilization, renal mobilization, splenectomy, distal pancreatectomy, cholecystectomy, and mesenteric lymphadenectomy. Nonetheless, distal pancreatectomy had an astonishing rate of pancreatic fistulas, which remains a major concern in this procedure [22]. Patients in the Whipple procedure group had a significantly longer hospital stay (14 ± 3.5 days) compared to the distal pancreatectomy group (11 ± 2.8 days, t = 2.25, p = 0.03), indicating a statistically significant difference, reflecting its complexity and increased postoperative management requirements. These findings align with previous studies, which reported a median hospital stay of 13-15 days for Whipple and 10-12 days for distal pancreatectomy [23]. Nevertheless, there was no perioperative mortality in either of the groups, and this is attributed to improvements in surgical procedures and their management. The results of the present study indicate the importance of the personalized approach to surgical management depending on the tumor location, patient, and disease factors [24]. The Whipple procedure is more effective for patients with pancreatic head cancers in terms of long-term survival, in return for increased postoperative complications and longer hospital stay. Distal pancreatectomy is safer and less complex; however, because of the aggressiveness of distal pancreatic adenocarcinomas, long-term survival gains may be diminished despite curative resections [25]. Recent advancements in minimally invasive techniques, including laparoscopic and robotic-assisted pancreatic surgery, have shown promise in reducing postoperative complications and hospital stays. Studies suggest that robotic-assisted Whipple procedures reduce hospital stays by two to three days and lower complication rates by 10% to 15% compared to open surgery [26]. Future studies should explore the impact of these approaches on long-term outcomes.

Limitations

This study has several limitations, including its small sample size (n=30) and retrospective design, which may introduce selection bias. The heterogeneity in tumor biology and stage at diagnosis could have influenced the outcomes. Additionally, missing data on tumor stage, margin status, and adjuvant therapy limit causal inferences, and single-center design limits generalizability. Future studies with larger patient cohorts and prospective designs are needed to validate these findings.

## Conclusions

Both the Whipple procedure and distal pancreatectomy are effective surgical options for pancreatic cancer, with their selection primarily dictated by tumor location. The Whipple procedure, performed for pancreatic head tumors, offers better long-term survival but carries a higher risk of complications, including delayed gastric emptying and pancreatic fistulas. In contrast, distal pancreatectomy, used for tumors in the body or tail, is associated with lower surgical complexity and fewer complications but may have less favorable survival outcomes due to the later-stage presentation of these tumors. Additionally, the frequent need for splenectomy in distal pancreatectomy increases the risk of postoperative infections.

Given the significant influence of tumor biology, stage, and patient factors on outcomes, a multidisciplinary approach is essential for optimal surgical decision-making. Future research should explore the role of minimally invasive techniques and improved perioperative management strategies to enhance patient outcomes in both procedures.
