# Correction: Whipple Procedure vs. Distal Pancreatectomy: A Study on the Efficacy, Survival Rates, and Complication Rates in Patients With Pancreatic Cancer

**DOI:** 10.7759/cureus.c401

**Published:** 2026-02-11

**Authors:** Hamza Siddique, Sabaina Arshad Tarar, Ahmed Salman Majeed, Muhammad Hamza, Mueez Saleem Raja, Muhammad Haider Naqvi, Muhammad Zarar, Fizza Chudhary, Bilal Qammar

**Affiliations:** 1 Internal Medicine, Social Security Hospital, Faisalabad, PAK; 2 General Surgery, Shalamar Hospital, Lahore, PAK; 3 Hepatobiliary and Liver Transplant Surgery, Pakistan Kidney and Liver Institute, Lahore, PAK; 4 General Surgery, Muzaffarabad General Hospital, Muzaffarabad, PAK; 5 Medicine and Surgery, Queen Mary University, London, GBR; 6 General Surgery, District Head Quarters (DHQ) Hospital, Faisalabad, PAK; 7 Emergency Medicine, Novocare International Hospital and Research Center, Sargodha, PAK

This article has been corrected as two sentences in the introduction section have been revised as follows:

Original: "Distal pancreatectomy, although technically easier than the Whipple procedure, carries risks such as postoperative pancreatic fistulas and splenic complications. Additionally, due to its proximity to the spleen, pancreatic pseudocysts are often associated with splenectomy. While the Whipple procedure is technically easier than the distal pancreatectomy, it has its risks too, which include postoperative pancreatic fistulas and splenic complications"

Corrected: "Distal pancreatectomy, although technically easier and less complex than the Whipple procedure, is still associated with risks such as postoperative pancreatic fistulas and splenic complications."

